# Effect of theophylline on serum and milk pharmacokinetics of tylosin following intramuscular administration in lactating goats

**DOI:** 10.1186/s12917-024-04089-6

**Published:** 2024-06-08

**Authors:** Fatma Sayed EL-Tareef, Khaled Abo-EL-Sooud, Mohamed Karmi, Ahmed Hafez

**Affiliations:** 1 Department of Pharmacology, Faculty of Veterinary Medicine, Aswan University, Aswan, Egypt; 2 Department of Pharmacology, Faculty of Veterinary Medicine, Cairo University, Giza, Egypt; 3 Department of Food Hygiene and Control, Faculty of Veterinary Medicine, Aswan, Egypt

**Keywords:** Pharmacokinetics, Tylosin, Theophylline, Goats, Milk

## Abstract

**Aim of the work:**

The study was conducted to evaluate the influence of theophylline pre-treatment on serum pharmacokinetics and milk elimination of tylosin following single intramuscular (IM) administrations in lactating goats.

**Methods and results:**

In a cross-over study, tylosin was injected via intramuscular (IM) at a single dose of 15 mg/kg b.wt. After a one-month washout period goats received theophylline at a daily IM dose of 2 mg/kg b.wt. for seven consecutive days then tylosin was injected IM dose of 15 mg/kg b.wt. two hours after the last theophylline dosing. Blood samples were collected before and at 0.25, 0.5, 0.75, 1, 2, 4, 6, 8, 10, 12, and 24 h post-injection. Samples were left to clot and then centrifuged to yield serum. Milk samples were collected before and at 0.5, 1, 2, 4, 6, 8, 10, 12, 24, 48, and 72 h post-injection from each goat by hand milking. Tylosin serum concentrations were determined by high-performance liquid chromatography (HPLC). Tylosin concentrations versus time were analyzed by a noncompartmental method. Tylosin C_max_ significantly declined from 1.73 ± 0.10 to 1.01 ± 0.11 µg/ml, and attained T_max_ values of 2 and 1 h, respectively in theophylline-pretreated goats. Moreover, theophylline pretreatment significantly shortened the elimination half-life (t_1/2el_) from 6.94 to 1.98 h, t_1/2ka_ from 0.62 to 0.36 h and the mean residence time (MRT) from 8.02 to 4.31 h, also Vz/F and AUCs decreased from 11.91 to 7.70 L/kg and from 12.64 to 4.57 µg*h/ml, respectively, consequently, theophylline enhanced the clearance (Cl/F) of tylosin from the body. Similarly, tylosin milk concentrations were significantly lower in theophylline-pretreated goats than in goats that received tylosin alone and were detected up to 24 and 72 h in both groups, respectively. Moreover, the t_1/2el_ and AUCs were significantly decreased from 14.68 ± 1.97 to 4.72 ± 0.48 h, and from 181 to 67.20 µg*h/ml, respectively.

**Conclusions:**

The withdrawal period for tylosin in goat milk is at least 72 h. Theophylline pretreatment significantly decreases serum and milk tylosin concentrations to subtherapeutic levels, which could have serious clinical consequences such as failure of therapy. This means that after administering tylosin to goats, milk from these animals should not be consumed for at least 96 h to ensure that the milk is free from residues of the antibiotic.

**Supplementary Information:**

The online version contains supplementary material available at 10.1186/s12917-024-04089-6.

## Introduction

Drug-drug interactions have become standard therapy for various complex diseases and the outcome may be intentional through improving therapeutic efficacy and preventing the development of monotherapy resistance [1]. Simultaneously, this type of interaction is mostly constituting growing problems in veterinary medicine [2] as it has a negative impact on the predicated drug effects. So, it is important to focus on this problem to understand how various medications interact when it is necessary to provide them concurrently [3]. Tylosin is a macrolide antibiotic that is exclusively permitted for veterinary usage. It acts as a bacteriostatic agent by attaching to the 50 S ribosomal subunit’s 23 S rRNA and preventing gram-positive bacteria from synthesizing proteins [4]. Tylosin has a powerful action against gram-positive bacteria, anaerobic bacteria, and mycoplasmas, which makes it useful for treating infections like swine streptococcosis, pneumonia, arthritis, and respiratory tract infections [5]. Moreover, tylosin is recommended for the treatment of mastitis caused by Gram-positive or mycoplasma spp. in small ruminants [6].

Tylosin is distributed widely across the body’s tissues and fluids because it has a high lipid solubility and a moderate affinity for plasma proteins [7]. Studies on the pharmacokinetics of tylosin have been conducted in a variety of species, including buffaloes, ewes, goats, cows [8–11]. In the European Union, some macrolides are approved for use in goats as tulathromycin. Given the limited FDA-approved medications for use in goats, drugs are commonly prescribed in an extra-label manner which is legalized by the Animal Medicinal Drug Use Clarification Act of 1994 (AMDUCA) [12]. Additionally, maximum residue limits (MRLs) have been extended from other species for tylosin in goats [13]. Macrolide antibiotics are used to treat a wide range of infections and are frequently combined with other classes of drug agents, increasing the prospect of pharmacokinetic interactions [14, 15].

Theophylline is a methylxanthine derivative, frequently used in veterinary medicine to treat bronchial asthma through inhibiting phosphodiesterase enzyme (PDE), stopping the breakdown of cAMP inside the bronchial smooth muscle and hereafter resulting in the dilation of the airways [16]. Also, theophylline has anti-inflammatory properties due to the inhibition of PDE4 and histone deacetylase-2 activation, resulting in switching off activated inflammatory genes [17]. Theophylline is a nonselective phosphodiesterase (PDE) inhibitor, and selective inhibitor of adenosine diphosphate (ADP) and platelet-activating factor (PAF) so inhibits the platelet aggregation in a concentration-dependent manner in equines [18]. Moreover, theophylline induced cardiovascular toxicity with acute hypotension in dogs [19]. Theophylline is mainly metabolized by different cytochrome P450 (CYP) subfamilies; CYP2E (hydroxylation) and CYP1A2 (N-demethylation) [20]. Some drugs are laboring as `probe agents’ for the study of the co-administered drug efficacy and pharmacokinetics. Earlier, the disposition kinetics of enrofloxacin was significantly altered with the concurrent `probe’ drug albendazole in goats [21]. In the present work, we compared the effect of the `probe’ drug, theophylline on the elimination of tylosin in goats. Species differences in the activity of drug-metabolizing enzymes in ruminants are encountered. In this respect, camels eliminate theophylline at a slower rate than goats, which provides via mixed function oxidases is significantly slower in camels than in goats [22]. Therefore, the influence of theophylline pretreatment on serum and milk pharmacokinetics of tylosin following IM injection in lactating goats is being investigated for the first time in this study.

## Materials and methods

### Methods

#### Ethics statement

All Management methods and care protocols were conducted per the guidelines of The Institutional Animal Care and Use Committee of the Faculty of Veterinary Medicine, Cairo University (Vet CU12/10/2021/335).

###  Experimental animals

Five healthy, non-pregnant native Baladi lactating goats, weighing from 22 to 35 kg and aged from 2 to 4 years were purchased from a private farm. During acclimatization (one month before starting the experiment to ensure the complete withdrawal of any residual drugs) and subsequent treatment periods, all goats were fed alfalfa and a concentrated diet with free access to water. The animals were kept indoors in an experimental animal shed.

###  Drugs

- Tylosin tartrate (Tylosin AVICO®, 200 mg tylosin tartrate/ml, AVICO Co) was used in this study. The high-performance liquid chromatography (HPLC) analytical grade acetonitrile, perchloric acid, trifluoroacetic acid, and tylosin reference standard were purchased from Sigma Aldrich Co.
- Theophylline (Minophylline -N® 5 ml ampoules (each 1 ml contains 25 mg aminophylline equivalent to 21.43 mg theophylline anhydrous) Alexandria Co).

###  Experimental design

In cross-over study goats were injected with tylosin intramuscularly into thigh muscles at a single dose of 15 mg/kg b.wt [23, 24]. After a one-month washout period to ensure clearance of goat’s bodies from any drug residues, goats were preinjected intramuscularly with theophylline (2 mg/kg b.wt. once daily) [22] for seven consecutive days, then tylosin was injected at a single IM dose of 15 mg/kg b.wt.

###  Samples collection

On the day first of injection, blood samples were collected before and at 0.25, 0.5, 0.75, 1, 2, 4, 6, 8, 10, 12, and 24 h post-injection. Samples were left to clot and then centrifuged to yield sera, milk samples were collected before and at 0.5, 1, 2, 4, 6, 8, 10, 12, 24, 48, and 72 h post-injection from each goat by hand milking and complete evacuation of the udder was performed after each sampling. In the second experiment, blood and milk samples were collected and processed as above after the injection of tylosin in theophylline-pretreated goats. All samples were stored at -80 °C until analysis.

###  Analysis of tylosin

Tylosin serum and milk concentrations were measured by high-performance liquid chromatography (HPLC) using the method previously described [25] with some modifications. The HPLC system (Shimadzu, Japan) consisted of a pump (model LC-10AS), manual injector, and photodiode array (PDA) detector connected to chromquest software. The chromatographic separation was performed using a Phenomenex hypersil C_18_ column (5 μm, 250 × 4.6 mm) with an isocratic mobile phase consisting of acetonitrile and water (30:70 v/v), and 0.5% of trifluoroacetic acid was added to the mobile phase. The mobile phase was filtered through a 0.45 μm filter and pumped into the column at a flow rate of 1.5 ml/min. The effluent was monitored at 287 nm. The retention time was 5.2 min. Frozen serum samples were thawed at room temperature, and 200 µl of serum was transferred to a centrifuge tube, where it was precipitated with 200 µl perchloric acid (8%). Each sample was mixed for 30 s with a vortex mixer before being centrifuged at 1500x g for 5 min. In the HPLC system, 50 µl of clear supernatant was introduced. The fat layer was separated from the milk with centrifugation for 10 min at 3000 rpm and 4 °C. Two ml of the lower skim layer was transferred to a new centrifuge tube, along with 4 ml of acetonitrile. The solution was combined and centrifuged at 3000 rpm and 4 °C for 10 min [26].

###  Method validation

The calibration curves of tylosin were prepared in free-drug samples with seven different concentrations between 0.025 and 10 µg/ml for serum and 0.07 and 20 µg/ml for milk. A calibration curve was obtained by plotting the peak area versus the nominal concentrations. The equation was calculated by the least-squares method using linear regression. The standard curve of tylosin in goats’ serum and milk was linear, laying between 0.025 and 5 µg/ml and 0.07 and 16 µg/ml, respectively. The coefficient of determination (*r*^2^) for the external standards for all the analytes ranged between 0.963 and 0.999. The peak area of the unknown specimen was compared with that of the standard tylosin. The precision and accuracy of the method were evaluated by repetitive analysis of the serum samples (*n* = 6) spiked with different known concentrations of tylosin. Intra-assay variations were determined by measuring seven replicates of three standard samples used for calibration curves. The intra-assay variation coefficients were < 6% for serum and milk. Inter-assay precision was determined by assaying the three standard samples on three separate days. The inter-assay variation coefficients were < 5% for serum and milk. Recovery of tylosin from serum and milk was found to be 97 and 95%, respectively. The Limit of tylosin detection (LOD) and limit of quantification (LOQ) for serum were 0.025 and 0.05 µg/ml and the LOQ for milk was 0.07 µg/ml.

###  Pharmacokinetic analysis

Serum concentrations of tylosin after IM were subjected to non-compartmental analysis based on the statistical moment theory using Add-in PKSolver Software, China Pharmaceutical University Version 2 [27]. After the IM administration, the following values were calculated: the terminal half-life (T_1/2el_) was calculated as 0.693/λz, elimination rate constant (λz, calculated as the slope of the terminal phase of the serum concentration curve that included a minimum of five points after the peak concentration). The peak plasma concentration (C_max_) and, the time needed to achieve C_max_, (T_max_) were determined directly from the serum concentration-time curve. The area under the serum concentration-time curve (AUC) and the area under the first moment curve (AUMC) were calculated using a linear trapezoidal rule. The mean residence time (MRT) = AUMC/AUC, serum clearance (Cl) = Dose/AUC, the apparent volume of distribution (Vz/F) was calculated as Cl/F/λz and relative bioavailability (F_rel_) is calculated by comparing the AUCs of tylosin alone and with theophylline [28].

###  Statistical analysis

The statistical analysis was performed using the SPSS®22.0 software. The obtained results were presented as mean ± standard deviation (SD). The concentrations and the pharmacokinetic parameters of tylosin in the absence and presence of theophylline were compared using the two-tailed Paired-Samples *t*-test. Means were considered significantly different at *p < 0.05.

##  Results

**Fig. 1:**
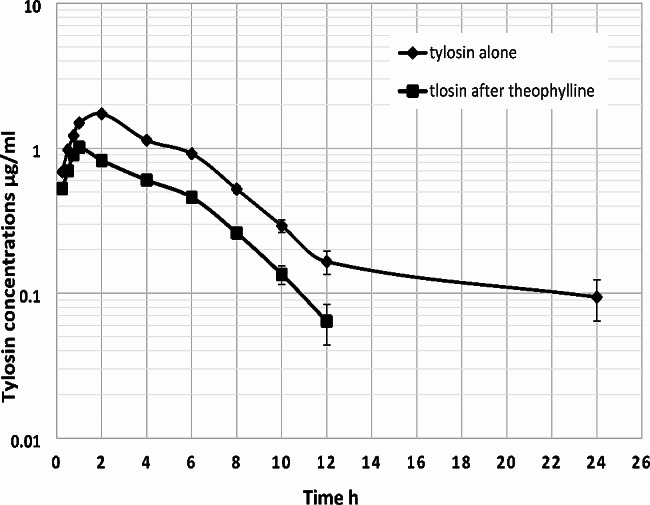
Mean ± SD of serum concentrations of tylosin (µg/ml) in tylosin alone and theophylline pretreated goats following intramuscular injection of 15 mg /kg b.wt. (*n* = 5)

The experimental goats showed no signs of toxicity or physical abnormalities after drug injections. Mean serum concentration-time curves following IM injection of tylosin alone and with theophylline pretreated-lactating goats are shown in Fig. 1. Pharmacokinetic parameters of tylosin following IM injection alone and with theophylline are presented in Table 1. Theophylline significantly decreased tylosin concentrations as the C_max_ decreased from 1.73 to 1.01 µg/ml. Moreover, theophylline pretreatment significantly shortened the elimination half-life from 6.94 to 1.98 h, and the MRT from 8.02 to 4.31 h, also the CL/F of tylosin was significantly increased from 1.19 to 2.73 L/h/kg, consequently, theophylline enhanced the elimination of tylosin from the body. There was a 56% decrease in AUC from 12.64 to 5.57 µg*h/ml in combination treatment and this seems to be associated with an increase in the elimination rate of tylosin in the presence of theophylline.

**Table 1:** Mean ± SD serum pharmacokinetic parameters of tylosin alone and theophylline pretreated goats following intramuscular administration of tylosin at 15 mg/kg b.wt. (*n* = 5)

Parameters	Unit	Tylosin alone	Tylosin with theophylline
λz	1/h	0.114 ± 0.05	0.354 ± 0.04*
Ka	1/h	1.12 ± 0.12	2.12 ± 0.21*
t_1/2 ka_	h	0.62 ± 0.07	0.33 ± 0.031*
t_1/2el_	h	6.94 ± 1.35	1.97 ± 0.196*
T_max_	h	2.00 ± 0.00	1.00 ± 0.00
C_max_	µg/ml	1.73 ± 0.10	1.01 ± 0.11*
AUC_0−∞_	µg*h/ml	12.64 ± 0.83	5.57 ± 0.78*
AUMC _0−∞_	µg*h^2^/ml	102 ± 24.06	24.128 ± 4.97
MRT_0−∞_	h	8.02 ± 1.75	4.31 ± 0.46*
Vz/F	L/kg	11.91 ± 0.91	7.70 ± 0.35*
Cl/F	L/h/kg	1.190 ± 0.08	2.73 ± 0.39*
F_Rel_	%	100	44

**Fig. 2:**
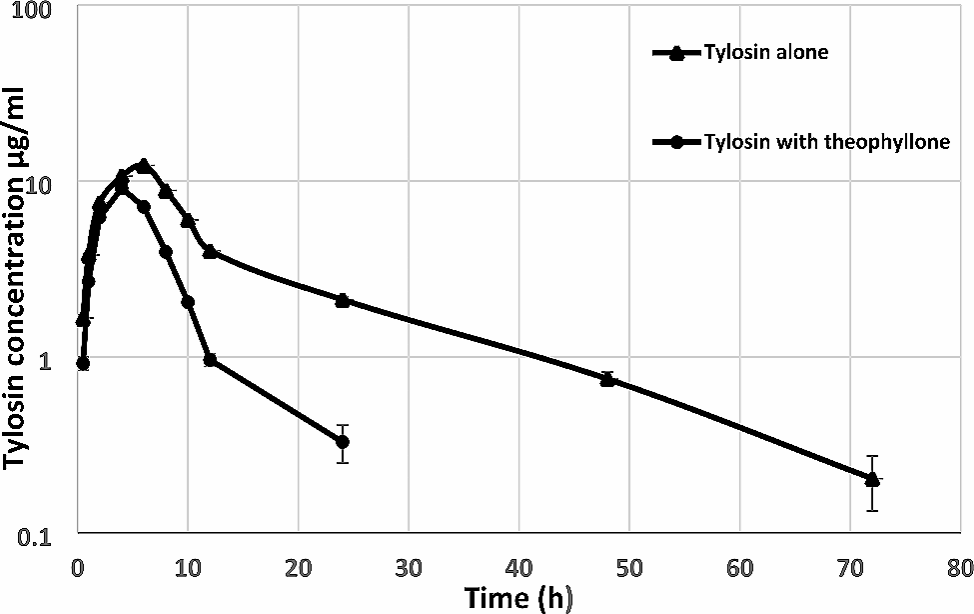
Mean ± SD milk concentrations (µg/ml) of tylosin alone and theophylline pretreated goats following intramuscular injection of 15 mg /kg b.wt. (*n* = 5)

Mean ± SD tylosin milk concentration-time curves alone and in theophylline pretreated goats following IM injection are shown in Fig. 2. Tylosin milk concentrations were significantly lower in goat’s milk pretreated with theophylline compared to tylosin alone and were detected up to 24 and 72 h in both groups, respectively. The pharmacokinetic parameters of milk are shown in Table 2. Tylosin penetrated milk from the blood quickly and extensively after being injected with IM, and its milk concentrations were higher than that in serum. However, theophylline pretreatment resulted in a significant decrease in tylosin concentrations in milk as the C_max_ values decreased from 12.24 to 9.08 µg/ml. The CL/F of tylosin was significantly increased from 0.09 ± 0.03 to 0.23 ± 0.05 L/h/kg while t_1/2el_ and MRT decreased significantly from 14.68 to 4.72 h, and 17.74 to 7.17 h, alone and theophylline pretreated goats, respectively. The volume of distribution (Vz/F) was not significantly changed.

**Table 2:** Mean ± SD milk pharmacokinetic parameters of tylosin alone and theophylline pretreated goats following intramuscular administration of tylosin at 15 mg/kg b.wt. (*n* = 5)

Parameters	Unit	Tylosin alone	Tylosin with theophylline
λz	1/h	0.048 ± 0.02	0.154 ± 0.03*
Ka	1/h	0.26 ± 0.14	0.34 ± 0.11
t_1/2 ka_	h	2.69 ± 0.34	2.04 ± 0.30
t_1/2el_	h	14.68 ± 1.97	4.72 ± 0.48*
T_max_	h	6.00 ± 0.00	4.00 ± 0.00
C_max_	µg/ml	12.24 ± 1.03	9.08 ± 0.88*
AUC_0−∞_	µg*h/ml	181 ± 23.34	67.20 ± 10.83*
AUC_milk_/AUC_serum_	Ratio	14.319	12.066
AUMC _0−∞_	µg*h^2^/ml	3203 ± 811	484 ± 107*
MRT_0−∞_	h	17.74 ± 2.10	7.17 ± 0.88*
Vz/F	L/kg	1.90 ± 0.25	1.51 ± 0.106
Cl/F	L/h/kg	0.09 ± 0.03	0.23 ± 0.05*

##  Discussion

Following IM administration, tylosin was detected from the 15th min up to 24 h after administration in the serum and from the 30th min up to 72 h after administration in the milk of goats. These results were found to be in similar to those obtained in goats [10] but the values were lower than those reported in cattle by Avci et al. [11] at higher dose (17.5 mg/kg). Theophylline significantly decreased serum concentration and tylosin was detected up to 12 h. The C_max_ of tylosin was 1.73 µg/ml and was attained at a T_max_ of 2 h. This is quite similar to those reported by Atef et al. [10] in goats at a dose of 10 mg/kg b.wt. IM (C_max_ 1.23 µg/ml at 1.96 h) and in cows [11] at a dose of 17.5 mg/kg b.wt. IM (C_max_ 1.3 µg/ml at 2 h). However, Taha et al. [24] showed a higher C_max_ of tylosin at a dose of 15 mg/kg b.wt. IM in Nubian goats (2.08 µg/ml at 3.83 h). This disparity may be attributed to the use of different commercial preparations and breeds of goats. Our present study revealed a significant decrease in tylosin serum concentration and AUC in theophylline-treated goats and these results agree with that of Paulsen et al. [29] who found significant decreases in the concentration and AUC of macrolide antibiotics with concomitant theophylline medication in humans as there is no previous data available in veterinary practice.

Similarly, Concurrent administration of ketotifen and theophylline is likely to reduce the therapeutic activities of both drugs and the concentration of theophylline increased with decrease in ketotifen concentration [30].

In the current study, the high tylosin volume of distribution (Vz/F) was 11.91 L/kg suggesting a wide diffusion in goat tissues. However, higher Vz/F of tylosin (20 L/kg) than our findings were observed in cows at a dose of 17.5 mg/kg b.wt. IM [11]. The wide tylosin Vz/F throughout the body may be attributed to both its high lipid solubility and moderate plasma protein binding. Thus, tylosin can reach the targeted site of infection at effective therapeutic concentrations, making it the drug of choice for combating systemic infection [10]. Thereafter, we found that the Vz/F of tylosin was lower in the pre-treated group (7.70 L/kg).

The mean elimination half-life (t_1/2el_) of tylosin alone was 6.94 h indicating a moderate rate of elimination of the drug. This is longer than the values previously reported for other animal species (calf at 2.24 h, buffalo at 2.40 h, sheep at 2.3 h, and pig at 3.01–3.88 h, ) [8, 9, 31]. Moreover, Avci et al. [11] showed a longer t_1/2el_ of tylosin (20.46 h) in cows. The variability in the elimination of half-lives of this antibiotic in different animal species is attributed to anatomical and physiological differences between these species as well as to the differences in drug formulation [9]. The prolonged elimination half-life of tylosin after IM injection in goats demonstrates that the concentration in the extravascular tissues is much higher than in the blood, a phenomenon common to macrolide antibiotics. These results comply with those reported in previous research on goats [23, 24] and sheep [9]. Theophylline induced some physiological changes such as an increase in liver blood flow [32], and this might have facilitated the hepatic metabolism biliary elimination of tylosin [33].

However, in theophylline pretreated goats tylosin was eliminated rapidly (t_1/2el_ 1.97 h), and there was a significant reduction in the tylosin serum concentrations from 15 min to 12 h. Also, tylosin cannot be detected after 12 h.

The systemic clearance (CL/F) of tylosin alone was 1.19 L/h/kg. This finding is lower than the value of CL/F (2.82 L/h/kg) reported in the duck [34]. Theophylline-pretreated goats cleared tylosin at a rapid rate (CL/F 2.73 L/h/kg). Consequently, the pharmacokinetic parameters (AUC, MRT, and t_1/2el_) were significantly decreased. So, we can illustrate that the lower serum concentration of tylosin, AUC, and shorter t_1/2el_ in goats pretreated with theophylline may be explained by the increased value of tylosin’s clearance.

In the present study, tylosin penetrated quickly and extensively into milk up to 72 h following treatment. These findings were consistent with those previously reported by [9] who investigated a distinct pattern that emerged, with detectable residual amounts identified in all animals up to 72 h following IM administration of a dose of 10 mg/kg b.wt in Najdi ewes. The tylosin concentrations in milk were significantly higher than the serum concentrations at all sampling intervals (*P* < 0.05). Drugs are generally accepted to cross the blood-milk barrier in the udder via non-ionic passive diffusion, and the extent of diffusion is greatly influenced by the drug’s physicochemical properties [35]. The thorough penetration of tylosin from the blood into goat’s milk of pH 6.6–6.8 was predictable based on an ion-trap mechanism which was reported for macrolide antibiotics including tylosin, due to its nature as a weak base (pKa = 7.1), renders milk tylosin unable to re-enter the general circulation [36]. Tylosin penetration into the milk was rapid and extensive with milk concentrations exceeding those in serum and greater than the minimal inhibitory concentrations for the majority of gram-negative udder pathogens were maintained for approximately 72 h. Moreover, tylosin has a large AUC_milk_/AUC_serum_ ratio of approximately 14:1, this finding is consistent to that reported to other macrolides in lactating goats [37]. Tylosin’s C_max_ and T_max_ values in the milk were found to be 12.24 µg/ml, and 6 h, respectively. In parallel previous studies demonstrated a maximum tylosin milk concentration of 6.68–7.41 µg/ml in sheep, 6.90 µg/ml in goats, and 6.22 µg/ml in cattle, and the time required to reach these concentrations as, 4.5–7 h, 6 h, and 6 h in the same animal species, respectively [9, 23, 38].

The t_1/2el_ of tylosin in milk was 14.68 h which is significantly shorter than reported (26.36 h) in cows [11]. The differences in the depletion of tylosin residue in milk could be ascribed to the distinct physiological differences that exist between goats and cows in the processes of milk secretion and milk composition.

Theophylline significantly decreased tylosin concentrations in milk, at the same time tylosin cannot be detected After 24 h. Also, the pharmacokinetic parameters C_max_, T_max_, AUC, MRT, and t_1/2el_ of tylosin in milk were significantly altered in theophylline-treated goats. While the clearance was significantly increased, and this may be the reason for the reduction in these parameters. Pharmacokinetic interaction of theophylline with tylosin decreased the tylosin concentration of which can result in decreased availability of tylosin at the target tissues and diminished its antibacterial efficacy.

In conclusion, the recommended withdrawal period for tylosin in goat milk is at least 72 h consequently, milk from tylosin-treated goats should not be consumed during this period to ensure that the milk is free from tylosin’s residues. Theophylline pretreatment significantly decreases serum and milk tylosin concentrations to subtherapeutic levels, which could have serious clinical consequences such as failure of therapy.

### Electronic supplementary material

Below is the link to the electronic supplementary material.

Supplementary Material 1

## Data Availability

The authors confirm that the data supporting the findings of this study are available within the article.

## Abbreviations

λz: terminal rate constant
Ka: absorption rate constant
t_1/2ka_: absorption half-life
t_1/2el_: elimination half-life
T_max_: time to the peak concentration
C_max_: maximum serum concentration
AUC_0−∞_: area under the curve from zero to infinity
AUMC_0−∞_: area under the first moment curve from zero to infinity
MRT_0−∞_: mean residence time from zero to infinity
Vz/F: apparent volume of distribution after extra-vascular route
Cl/F: clearance of drug after extra-vascular route
F_Rel_: relative bioavailability
HPLC: High-performance liquid chromatography
AMDUCA: Animal Medicinal Drug Use Clarification Act
MRLs: Maximum residue limits
LOD: limit of detection
LOQ: limit of quantification
