# Supplementary figures and images for: Effect of theophylline on serum and milk pharmacokinetics of tylosin following intramuscular administration in lactating goats
Source: BMC Vet Res. 2024 Jun 8;20:251. doi: 10.1186/s12917-024-04089-6 (PMC11162035; doi:10.1186/s12917-024-04089-6)

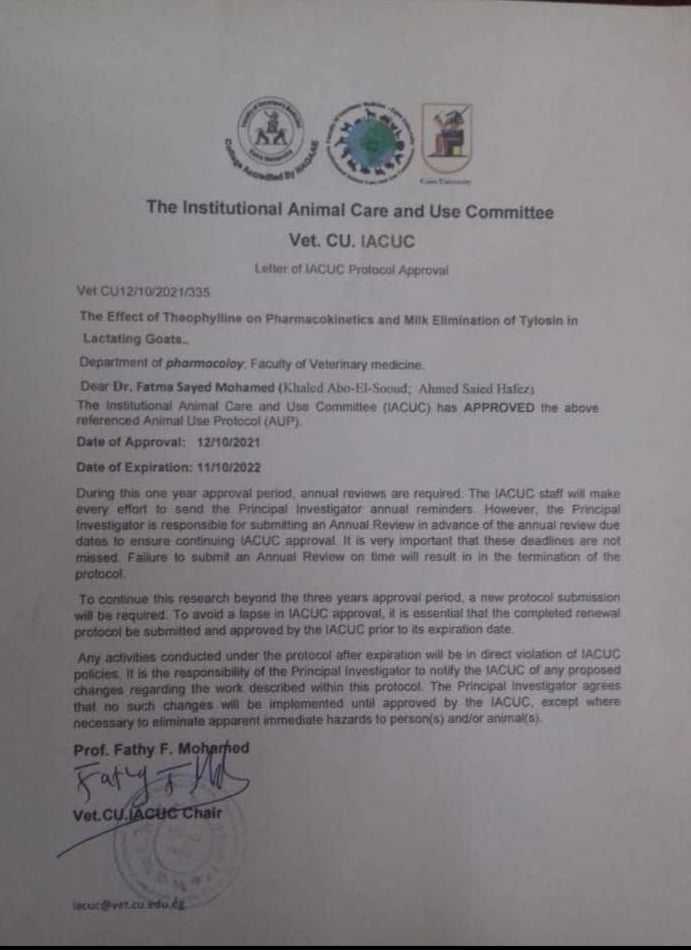

Supplement: Supplementary file 1 — Supplementary Material 1 [file 12917_2024_4089_MOESM1_ESM.jpg]
